# Multimodal Interventions for Chronic Low Back Pain: A Pilot Randomized Trial of Acupuncture, Stretching, and Qigong

**DOI:** 10.7759/cureus.91535

**Published:** 2025-09-03

**Authors:** Spyridon Sotiropoulos, Andreas Mavrogenis, Maria Papandreou, George Georgoudis

**Affiliations:** 1 Department of Physiotherapy, Musculoskeletal Physiotherapy Research Laboratory, University of West Attica (UNIWA), Athens, GRC; 2 Department of Orthopaedics, School of Medicine, National and Kapodistrian University of Athens, Athens, GRC; 3 Department of Physiotherapy, University of West Attica (UNIWA), Athens, GRC

**Keywords:** acupuncture therapy, chronic low back pain (clbp), disability, pilot study, pressure pain threshold (ppt), psychosocial outcomes, qigong, stretching exercises

## Abstract

Introduction: Chronic low back pain (cLBP) is a leading cause of disability worldwide. Acupuncture has demonstrated benefits in cLBP management. Combining it with stretching or Qigong may enhance therapeutic outcomes. This pilot study assessed the feasibility and preliminary effects of acupuncture alone and in combination with stretching or Qigong.

Methods: Thirty participants with cLBP were randomly assigned to receive (A) acupuncture only, (B) acupuncture plus static stretching, or (C) acupuncture plus modified Qigong, over eight weeks. The primary outcome was the pressure pain threshold (PPT). Secondary outcomes included pain intensity, disability, physical function, psychosocial measures, and quality of life. Outcomes were assessed at baseline, post-intervention, and two-month follow-up. Data were analysed using linear mixed-effects models. However, the study was not powered to detect between-group changes, as the aim of the study was to investigate the feasibility of the design, and the results need to be interpreted with caution.

Results: All groups showed statistically significant improvements over time in PPT, pain intensity, disability, functional performance, psychosocial status, and physical quality of life (p < 0.001). No significant group-by-time interactions were observed. However, descriptive trends suggested greater improvements in the PPT and function in the combination therapy groups. Several outcomes exceeded minimal clinically important differences, and large effect sizes were observed for pain (d = −3.32), disability (d = −1.85), and psychosocial distress (d = −1.17). No adverse events occurred, and adherence was high.

Conclusions: Acupuncture, alone and in combination with stretching or Qigong, resulted in clinically meaningful improvements in pain sensitivity, disability, and psychosocial outcomes. The protocol was feasible and well-tolerated. There are emerging indications from this study that combining acupuncture with other modalities may enhance therapeutic effects, highlighting the need for a larger sample to confirm these findings.

## Introduction

Chronic low back pain (cLBP) is one of the most common and disabling musculoskeletal disorders globally, with a point prevalence of 11.9% and a one-month prevalence of 23.2%, particularly affecting women and adults aged 40-80 years [1]. Despite a variety of treatment options, a significant proportion of individuals experience persistent symptoms, emphasizing the need for more effective and integrated management strategies [2].

Recent guidelines advocate for non-pharmacological, multimodal interventions to address the complex, biopsychosocial mechanisms underlying cLBP, including nociplastic pain, central sensitization, and fear-avoidance behaviors [3,4]. Acupuncture is effective for reducing pain and improving function in cLBP [5]. Stretching exercises can alleviate mechanical loading and restore movement patterns disrupted in cLBP and are proposed as a treatment option for cLBP [6]. Furthermore, there is growing evidence that Qigong, a mind-body practice integrating breathing, movement, and attention, has been shown to improve pain intensity, mood, and disability in musculoskeletal pain populations [7,8]. Although each of these interventions is supported by evidence, there are still no studies evaluating the combined effects of acupuncture with either stretching or Qigong. This represents a notable gap, as such combinations may offer additive or synergistic effects through multimodal targeting of musculoskeletal, neurological, and psychosocial domains.

This pilot randomized controlled trial aimed to explore the feasibility and preliminary effects of three interventions, acupuncture alone, acupuncture combined with stretching, and acupuncture combined with Qigong, on pain sensitivity, pain-related disability, and psychosocial outcomes in adults with CLBP. Findings will inform the development of integrated, person-centered care strategies and guide the design of a future definitive trial.

## Materials and methods

Study design

This was a three-arm, parallel-group, randomized pilot trial, conducted following the CONSORT 2010 guidelines for randomized trials, comparing (1) acupuncture alone, (2) acupuncture combined with static stretching, and (3) acupuncture combined with modified Qigong exercise in patients with cLBP [9]. The study aimed to explore the preliminary effects of these interventions and inform the design of a future full-scale randomized controlled trial. The trial was registered with the ISRCTN registry under ID ISRCTN15963005, accessible at https://www.isrctn.com/ISRCTN15963005.

Participants were adults aged 18-65 years with cLBP lasting longer than three months and reporting pain intensity greater than 5 on a Numerical Rating Scale (NRS, 0-10). Exclusion criteria included participation in physiotherapy or structured exercise within the past three months, diagnosis of specific spinal pathologies (e.g., malignancy, fractures, inflammatory conditions), recent spinal surgery, pregnancy or lactation, or the presence of red flag symptoms.

Participants were recruited from the Pain and Palliative Medicine Outpatient Clinic of the First University Department of Anesthesiology at Aretaieio Hospital, Athens. The study was conducted in accordance with the Declaration of Helsinki and approved by the Research Ethics and Deontology Committee of Aretaieio Hospital, National and Kapodistrian University of Athens (Approval Protocol Number: 377/15-11-2021). All participants provided written informed consent prior to participation.

This pilot study included 30 participants, randomly assigned to one of three groups (n = 10 per group). A formal sample size calculation was not conducted, as the aim was to explore trends and assess feasibility for a future definitive trial.

Randomization and blinding

**Figure 1:**
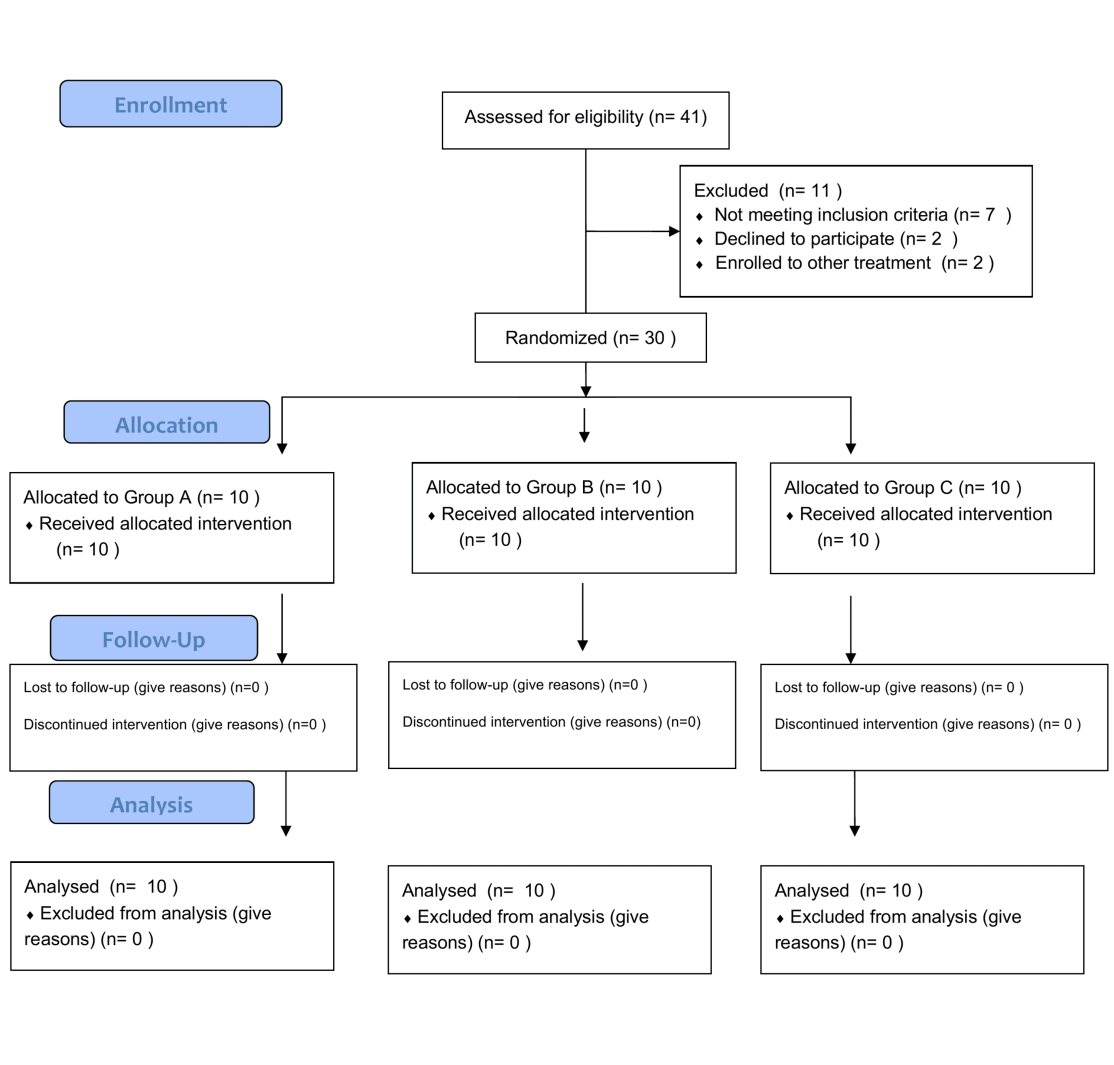
CONSORT 2010 Flow Diagram Illustrating Participant Progression through the Study Phases, Including Enrollment, Allocation, Follow-up, and Analysis. CONSORT: Consolidated Standards of Reporting Trials

Participants were randomly assigned to one of three groups (A, B, or C) using a simple randomization method with allocation concealment via opaque envelopes. As the sample was drawn from a relatively homogeneous clinic population, this approach was considered acceptable for a pilot trial, despite the potential risk of group imbalance. Group allocation was conducted by physicians at the Pain and Palliative Medicine Clinic, who were unaware of the specific interventions associated with each group, ensuring allocation concealment. Outcome assessors remained blinded to group assignment throughout the study. Due to the nature of the interventions, participants were aware of their participation in exercise interventions but were blinded to their group assignment and the study hypotheses. Participants' flow in the trial is depicted in Figure 1. 

Interventions

Group A: Acupuncture Only

Participants received a standardized acupuncture protocol based on STRICTA recommendations and WHO acupuncture point locations [10]. Sixteen core points were used (LI4, ST36, SP6, GB30, BL23-26), with bilateral electroacupuncture applied at BL23 and BL26 at 2Hz, to the degree tolerated by the participant. Sterile, single-use, stainless steel acupuncture needles (DongBang Acupuncture Inc., South Korea) were used for all treatments. Most points were needled with 0.30 × 40 mm needles, except for LI4, which was needled with 0.25 × 25 mm needles. In addition, the therapist could select up to four individualized points based on clinical examination. All sessions were administered by the same trained acupuncturist and lasted 25 minutes.

Group B: Acupuncture & Stretching Exercises

Participants in Group B received the same acupuncture treatment as Group A. They also followed a prescribed stretching program targeting key musculature relevant to low back pain (Appendix A) designed in accordance with the FITT principle. The routine included double knee to chest, single knee to chest, hamstring stretch, piriformis stretch, tensor fascia lata stretch, abdominal stretch, cat-camel stretch, slump stretch, and calf stretch. Each stretch was held for 30 seconds, repeated three times with 30 seconds of rest. Exercises were performed under supervision twice weekly for the first four weeks, and once weekly for the next four weeks. Each session lasted approximately 15-20 minutes. Participants were instructed to perform the routine daily at home following printed illustrated instructions.

Group C: Acupuncture & Qigong

Participants in this group received the same acupuncture protocol as the other two groups. Additionally, they participated in a supervised modified Baduanjin Qigong program based on protocols used in clinical rehabilitation research (see Appendix B). The eight-movement routine was practiced under the supervision of a trained physiotherapist twice weekly for the first four weeks and then once weekly for four additional weeks. Each session lasted approximately 15-20 minutes. Participants were instructed to practice daily at home using printed materials. All interventions lasted for eight weeks.

Outcome measures

All outcomes were assessed at three distinct time points: baseline (T1), immediately following the eight-week intervention (T2), and at a two-month follow-up after the completion of the intervention (T3). Assessments were conducted in a single session per time point, following a standardized order: questionnaires were completed first, in a random order, followed by pressure algometry and physical function testing. To ensure consistency, sessions were scheduled at similar times of day for each participant. All outcomes were assessed by evaluators who were blinded to group allocation.

The primary outcome was the pressure pain threshold (PPT), assessed using the Commander Echo Algometer (JTECH Medical, Midvale, UT, USA), a handheld digital algometer with a 1 cm² rubber-tipped probe (JTECH Medical, 2023). The PPT was measured at nine anatomical sites: the spinous process of L3 with the participant prone; bilateral paraspinal muscles at L4 and L5, located 2 cm lateral to the spinous processes over the erector spinae belly, also with the participant prone; bilateral gluteus medius, identified at the midpoint between the iliac crest and greater trochanter, with the participant side-lying; bilateral tibialis anterior, located one-third of the distance from the tibial tuberosity to the lateral malleolus, with the participant supine; and bilateral upper trapezius, located at the midpoint between the acromion and the spinous process of C7, with the participant seated (see Appendix C).

Pressure was applied perpendicularly at a constant rate of 1 kg/cm² per second. Participants were instructed to verbally indicate the transition from pressure to pain. Three consecutive measurements were recorded at each site, with a 30-second rest interval between trials. The mean of the three readings was used for analysis [11,12]. Previous studies have demonstrated reliable PPT measurement at all selected sites, which were re-marked at each session to ensure consistency [11,13]. The evaluator remained blinded to group allocation.

Secondary outcomes included self-reported pain intensity, measured by the Short Form McGill Pain Questionnaire (SF-MPQ) [14,15]. Functional status was evaluated using the Roland Morris Disability Questionnaire (RMDQ) [16,17] and the Five-Repetition Sit-to-Stand Test (5RSTS) [18]. Psychosocial factors were assessed with the Fear Avoidance Beliefs Questionnaire (FABQ) [19,20], the Hospital Anxiety and Depression Scale (HADS) [21,22], and the Pain Catastrophizing Scale (PCS) [23,24]. Health-related quality of life was measured using the 12-Item Short Form Health Survey (SF-12) [25,26], and overall patient-perceived improvement was recorded using the Global Perceived Effect (GPE) scale [27]. Adherence to the prescribed exercise programs (stretching and Qigong) was monitored through participant-completed daily diaries, which were reviewed at each follow-up session. Blank entries were considered non-adherent days. Adherence percentages were calculated as the number of completed days divided by the total number of prescribed days. Missing or unticked days in the diary were treated conservatively and assumed to reflect days when the exercises were not performed, to avoid overestimating adherence. All questionnaires were administered using validated Greek-language versions, and permission for their use was obtained where needed.

Statistical analysis

Descriptive statistics (means, standard deviations, frequencies) were used to summarize baseline characteristics. Normality was assessed using the Shapiro-Wilk test and visual inspection of histograms to determine the use of parametric or non-parametric tests. Longitudinal changes were evaluated using a linear mixed-effects model for repeated measures (MMRM), with fixed effects for group, time (categorical: baseline, post-intervention, follow-up), and their interaction. Participant ID was included as a random intercept. Models were estimated using maximum likelihood without small-sample correction (e.g., Kenward-Roger), in line with exploratory pilot study recommendations [28,29]. Residuals were examined using Q-Q and residual-versus-fitted plots to assess normality and homoscedasticity. For selected outcomes, repeated measures ANOVA was also performed to examine group-by-time interactions, main effects of time, and between-group differences. Effect sizes (Cohen’s d) were calculated from raw mean differences and pooled standard deviations; model-based estimates were not used. Multiple comparison adjustments were not applied, consistent with methodological guidance for feasibility studies prioritizing signal detection over confirmatory inference [30]. All analyses were conducted using Stata v17.0 (MMRM; StataCorp LLC, College Station, Texas, USA) and IBM SPSS Statistics for Windows, Version 28 (Released 2021; IBM Corp., Armonk, New York, United States). No data were missing; therefore, no imputation was necessary.

## Results

Participant characteristics

All 30 participants completed the study (n=24, 80% female; mean age 49 ± 17 years) with no dropouts or adverse events. Baseline demographic and clinical characteristics are presented in Table 1, and anthropometric characteristics in Table 2. Most participants had cLBP for more than one year (n=19, 63.3%), and approximately half reported regular use of analgesic or adjunctive medications.

**Table 1:** Baseline Demographic and Clinical Characteristics Data presented as count (N) and percentage (%). Group A: Acupuncture + Stretching; Group B: Acupuncture + Qigong; Group C: Control.

		Group A		Group B		Group C		Total	
		N	N%	N	N%	N	N%	N	N%
Gender	Male	3	30%	2	20%	1	10%	6	20%
	Female	7	70%	8	80%	9	90%	24	80%
Age Group	18-29	1	10%	2	20%	4	40	7	23.3%
	30-39	0	0%	0	0%	1	10%	1	3.3%
	40-49	0	0%	1	10%	1	10%	2	6.7%
	50-59	2	20%	5	50%	1	10%	8	26.7%
	60-65	7	70%	2	20%	3	20%	12	40%
Symptoms duration (months)	3-6	3	30%	2	20%	4	40%	9	30%
	6-12	0	0%	0	0%	2	20%	2	6.7%
	12-24	0	0%	0	0%	1	10%	1	3.3%
	>24m	7	70%	8	80%	3	30%	18	60%

**Table 2:** Anthropometric Characteristics Values are presented as mean ± standard deviation (SD). Group A: Acupuncture + Stretching; Group B: Acupuncture + Qigong; Group C: Control. All groups included 10 participants.

Measure	Group A (N=10) Mean	SD	Group B (N=10) Mean	SD	Group C (N=10) Mean	SD	Total (N=30) Mean	SD
Age	57	13	49	15	41	19	49	17
Height (m)	1.67	0.10	1.67	0.10	1.66	0.09	1.67	0.09
Weight (kg)	80.0	13.7	74.0	10.1	61.5	7.6	71.8	13.0

Primary outcome: PPT

PPTs increased significantly over time across all anatomical sites, with a consistent main effect of time observed in all models (p < 0.001). Group-by-time interaction p-values ranged from 0.010 to 0.291, indicating statistically significant differences in temporal response at specific sites. Between-group effects, irrespective of time, were not significant overall, with p-values ranging from 0.176 to 0.986. Effect sizes (Cohen’s d) ranged from 0.77 to 1.05, reflecting moderate to large improvements in pressure pain sensitivity across the sample.

Mixed-effects post hoc contrasts identified significant between-group differences at selected sites and time points. At the L3 spinous process, Group B demonstrated a significantly greater increase in PPT at follow-up compared to Group A (b = −2.944, SE = 0.797, z = −3.69, p < 0.001). At the L4 left site, Group B had significantly higher PPT at post-intervention than Group A (b = −2.480, SE = 0.727, z = −3.41, p = 0.001). Additional significant differences were observed at the L4 right site at follow-up (p = 0.019) and at the left tibialis anterior at follow-up (p = 0.032). No significant differences were observed between Groups A and C or between Groups B and C.

Site-level summary statistics and model results are presented in Table 3.

**Table 3:** Mean Pressure Pain Thresholds (PPT, kg/cm²) Across Time Points by Anatomical Site and Treatment Group This table presents pressure pain thresholds (PPT), measured in kilograms per square centimeter (kg/cm²), recorded at baseline (T1), post-intervention (T2), and two-month follow-up (T3) for each anatomical site and intervention group. A: Group A, B: Group B, C: Group C Values are reported as means with standard deviations (SD) and 95% confidence intervals (CI). P-values correspond to fixed effects from linear mixed-effects models evaluating the effects of time, group, and the group-by-time interaction. Cohen’s d represents the pooled effect size from baseline to follow-up. Anatomical site labels are defined as follows: L3 refers to the spinous process of the third lumbar vertebra. L4 and L5 refer to the paraspinal muscles at the respective lumbar levels, located 2 cm lateral to the spinous process. Gl. M. indicates the gluteus medius muscle. Tib. Ant. refers to the tibialis anterior muscle. Trap. indicates the upper trapezius muscle. L and R denote left and right sides, respectively. Level of significance p<0.05

Site	Group A: n=10, Group B: n=10, Group C: n=10	T1 Mean SD	T1 95% CI	T2 Mean SD	T2 95% CI	T3 Mean SD	T3 95% CI	p-value of interaction (group*time)	p-value for the overall trend over time (no grouping)	p-value for difference between groups (regardless of the time measured)	Cohen's d (effect size)
L3	A	5.40 ± 2.00	[3.97, 6.83]	7.40 ± 1.80	[6.11, 8.69]	8.34 ± 2.25	[6.73, 9.95]	0.01	<0.001	0.314	0.85
	B	7.1 ± 2.2	[5.53, 8.67]	6.60 ± 2.00	[5.17, 8.03]	7.17 ± 1.54	[6.07, 8.27]				
	C	5.2 ± 1.2	[4.34, 6.06]	6.20 ± 0.90	[5.56, 6.84]	7.08 ± 1.65	[5.90, 8.26]				
L4 Left	A	5.8 ± 1.8	[4.51, 7.09]	8.30 ± 1.70	[7.08, 9.52]	7.90 ± 2.20	[6.33, 9.47]	0.027	<0.001	0.215	0.87
	B	7.30 ± 2.00	[5.87, 8.73]	7.40 ± 1.80	[6.11, 8.69]	8.40 ± 2.10	[6.90, 9.90]				
	C	5.00 ± 1.80	[3.71, 6.29]	6.70 ± 1.90	[5.34, 8.06]	7.40 ± 2.30	[5.75, 9.05]				
L4 Right	A	5.30 ± 1.70	[4.08, 6.52]	8.20 ± 1.70	[6.98, 9.42]	8.30 ± 2.30	[6.65, 9.95]	0.019	<0.001	0.346	0.99
	B	6.70 ± 2.00	[5.27, 8.13]	7.50 ± 1.60	[6.36, 8.64]	8.10 ± 2.10	[6.60, 9.60]				
	C	5.50 ± 2.00	[4.07, 6.93]	6.50 ± 1.70	[5.28, 7.72]	7.20 ± 2.10	[5.70, 8.70]				
L5 Left	A	5.40 ± 1.90	[4.04, 6.76]	7.60 ± 1.90	[6.24, 8.96]	7.80 ± 2.20	[6.23, 9.37]	0.094	<0.001	0.703	1.05
	B	6.50 ± 1.70	[5.28, 7.72]	7.00 ± 1.40	[6.00, 8.00]	8.00 ± 2.00	[6.57, 9.43]				
	C	5.40 ± 1.90	[4.04, 6.76]	6.70 ± 1.40	[5.70, 7.70]	7.60 ± 1.80	[6.31, 8.89]				
L5 Right	A	5.70 ± 2.00	[4.27, 7.13]	7.80 ± 2.00	[6.37, 9.23]	7.80 ± 2.20	[6.23, 9.37]	0.076	<0.001	0.476	0.80
	B	6.90 ± 2.50	[5.11, 8.69]	7.00 ± 1.30	[6.07, 7.93]	7.70 ± 2.10	[6.20, 9.20]				
	C	5.10 ± 1.90	[3.74, 6.46]	6.70 ± 1.50	[5.63, 7.77]	7.30 ± 2.10	[5.80, 8.80]				
Gl. M. L	A	4.60 ± 2.00	[3.17, 6.03]	6.40 ± 1.60	[5.26, 7.54]	6.40 ± 2.10	[4.90, 7.90]	0.212	<0.001	0.651	0.79
	B	5.60 ± 2.40	[3.88, 7.32]	6.00 ± 1.70	[4.78, 7.22]	6.40 ± 2.00	[4.97, 7.83]				
	C	3.90 ± 1.40	[2.90, 4.90]	5.80 ± 1.70	[4.58, 7.02]	6.20 ± 2.10	[4.70, 7.70]				
Gl. M. R	A	5.10 ± 2.40	[3.38, 6.82]	7.10 ± 2.00	[5.67, 8.53]	6.60 ± 2.00	[5.17, 8.03]	0.193	<0.001	0.277	0.80
	B	5.50 ± 2.30	[3.85, 7.15]	5.80 ± 1.50	[4.73, 6.87]	6.50 ± 2.00	[5.07, 7.93]				
	C	3.70 ± 1.10	[2.91, 4.49]	5.80 ± 2.00	[4.37, 7.23]	6.00 ± 1.80	[4.71, 7.29]				
Tib. Ant. L	A	5.40 ± 1.90	[4.04, 6.76]	7.50 ± 2.30	[5.85, 9.15]	7.20 ± 2.40	[5.48, 8.92]	0.032	<0.001	0.860	0.91
	B	6.40 ± 2.00	[4.97, 7.83]	6.70 ± 1.40	[5.70, 7.70]	8.10 ± 2.00	[6.67, 9.53]				
	C	5.50 ± 1.90	[4.14, 6.86]	8.10 ± 2.10	[6.60, 9.60]	7.60 ± 2.10	[6.10, 9.10]				
Tib. Ant. R	A	5.70 ± 1.80	[4.41, 6.99]	7.70 ± 2.10	[6.20, 9.20]	7.40 ± 2.20	[5.83, 8.97]	0.188	<0.001	0.986	0.85
	B	6.50 ± 2.00	[5.07, 7.93]	6.80 ± 2.00	[5.37, 8.23]	7.80 ± 2.50	[6.01, 9.59]				
	C	5.70 ± 1.70	[4.48, 6.92]	7.70 ± 1.90	[6.34, 9.06]	7.70 ± 1.70	[6.48, 8.92]				
Trap. L	A	4.20 ± 1.90	[2.84, 5.56]	5.30 ± 1.80	[4.01, 6.59]	4.90 ± 1.40	[3.90, 5.90]	0.144	<0.001	0.176	0.77
	B	3.90 ± 0.90	[3.26, 4.54]	4.30 ± 0.80	[3.73, 4.87]	5.20 ± 1.50	[4.13, 6.27]				
	C	2.90 ± 1.30	[1.97, 3.83]	4.00 ± 1.40	[3.00, 5.00]	4.20 ± 1.60	[3.06, 5.34]				
Trap. R	A	4.20 ± 2.00	[2.77, 5.63]	4.70 ± 1.60	[3.56, 5.84]	4.90 ± 1.50	[3.83, 5.97]	0.291	<0.001	0.189	0.79
	B	4.00 ± 0.80	[3.43, 4.57]	4.50 ± 1.00	[3.78, 5.22]	5.10 ± 0.90	[4.46, 5.74]				
	C	2.80 ± 1.00	[2.08, 3.52]	4.10 ± 1.40	[3.10, 5.10]	4.20 ± 1.40	[3.20, 5.20]				

Pain intensity, disability, and functional performance

All four outcomes, Visual Analogue Scale (VAS), SF-MPQ, RMDQ, and the 5RSTS, demonstrated significant improvements over time across all groups (p < 0.001 for time in all models). Group-by-time interaction p-values ranged from 0.296 to 0.924, indicating no statistically significant differential group effects over time. Between-group differences, regardless of time, were not significant (p-values ranging from 0.236 to 0.931). Effect sizes ranged from −0.57 to −3.32, reflecting moderate to large improvements in pain, disability, and performance measures from baseline to follow-up.

Post hoc contrasts from the mixed-effects models confirmed that reductions in VAS and SF-MPQ scores were significant at both post-intervention and follow-up compared to baseline, with no significant between-group differences at either time point. For the RMDQ and 5RSTS, time-based effects were significant across all groups, but no group comparisons reached statistical significance at any time point. Summary statistics, confidence intervals, and model results are presented in Table 4.

**Table 4:** Visual Analogue Scale (VAS), Short-Form McGill Pain Questionnaire (SF-MPQ), Roland-Morris Disability Questionnaire (RMDQ), and Five-Repetition Sit-to-Stand Test (5RSTS) Scores Across Time Points by Treatment Group This table presents scores for the VAS, SF-MPQ, RMDQ, and 5RSTS at three time points: baseline (T1), post-intervention (T2), and two-month follow-up (T3), by the intervention group. A: Group A, B: Group B, C: Group C. Values are depicted as means with standard deviations (SD) and 95% confidence intervals (CI). p-values refer to fixed effects from linear mixed-effects models for group, time, and group-by-time interaction. Cohen’s d represents the effect size from baseline to follow-up. Level of significance p<0.05

Outcome	Group	T1 Mean SD	T1 95% CI	T2 Mean SD	T2 95% CI	T3 Mean SD	T3 95% CI	p-value of interaction (group*time)	p-value for the overall trend over time (no grouping)	p-value for the difference between groups (regardless of the time measured)	Cohen's d (effect size)
VAS	A	7.00 ± 1.05	[6.25, 7.75]	3.10 ± 1.60	[1.96, 4.24]	2.30 ± 1.16	[1.47, 3.13]	0.924	<0.001	0.236	-3.32
	B	7.50 ± 1.43	[6.48, 8.52]	3.90 ± 1.73	[2.66, 5.14]	2.80 ± 1.62	[1.64, 3.96]				
	C	6.50 ± 1.18	[5.66, 7.34]	2.70 ± 1.95	[1.30, 4.10]	2.10 ± 1.73	[0.86, 3.34]				
SF-MPQ	A	20.70 ± 8.67	[14.50, 26.90]	12.00 ± 7.42	[6.69, 17.31]	7.40 ± 5.76	[3.28, 11.52]	0.442	<0.001	0.856	-1.93
	B	22.50 ± 6.49	[17.86, 27.14]	10.30 ± 7.53	[4.91, 15.69]	6.80 ± 4.26	[3.75, 9.85]				
	C	18.60 ± 9.12	[12.08, 25.12]	9.60 ± 9.11	[3.08, 16.12]	7.40 ± 7.11	[2.32, 12.48]				
RMDQ	A	10.50 ± 3.89	[7.72, 13.28]	5.80 ± 3.52	[3.28, 8.32]	3.50 ± 3.03	[1.33, 5.67]	0.296	<0.001	0.931	-1.85
	B	12.00 ± 4.37	[8.87, 15.13]	4.50 ± 4.48	[1.30, 7.70]	3.50 ± 3.78	[0.80, 6.20]				
	C	9.60 ± 5.06	[5.98, 13.22]	5.30 ± 5.56	[1.32, 9.28]	3.40 ± 3.50	[0.89, 5.91]				
5RSTS	A	18.47 ± 15.80	[7.16, 29.78]	13.42 ± 12.74	[4.31, 22.53]	12.29 ± 12.08	[3.65, 20.93]	0.629	<0.001	0.401	-0.57
	B	14.02 ± 2.73	[12.07, 15.97]	10.08 ± 1.99	[8.66, 11.50]	9.70 ± 1.85	[8.38, 11.02]				
	C	12.46 ± 3.23	[10.15, 14.77]	9.10 ± 2.49	[7.32, 10.88]	8.66 ± 2.15	[7.12, 10.20]				

Psychosocial outcomes

Psychosocial measures, including the PCS, FABQ, and the HADS, showed significant improvements over time across all groups (p < 0.001 for the main effect of time). Group-by-time interaction p-values ranged from 0.375 to 0.874, indicating no significant differences in change trajectories between intervention groups. Between-group effects, regardless of time, were not statistically significant (p-values ranging from 0.291 to 0.866). Effect sizes (Cohen’s d) ranged from −0.67 to −1.18, representing moderate to large reductions in pain-related psychological and behavioral responses.

PCS and FABQ scores were significantly reduced at both post-intervention and follow-up compared to baseline, with no significant between-group differences at either time point. For both the anxiety and depression subscales of the HADS, time-based effects were significant across all groups, but no group comparisons reached statistical significance at any time point. Summary statistics, confidence intervals, and model results are presented in Table 5.

**Table 5:** Pain Catastrophizing Scale (PCS), Fear-Avoidance Belief Questionnaire (FABQ), and Hospital Anxiety and Depression Scale (HADS) Scores Across Time Points by the Treatment Group This table presents scores for the Pain Catastrophizing Scale (PCS), Fear-Avoidance Beliefs Questionnaire (FABQ), and Hospital Anxiety and Depression Scale (HADS—Anxiety and Depression subscales) measured at baseline (T1), post-intervention (T2), and two-month follow-up (T3), stratified by intervention group. A: Group A, B: Group B and C: Group C. All values are reported as means with standard deviations (SD) and 95% confidence intervals (CI). p-values represent fixed effects from linear mixed-effects models evaluating the influence of time, group, and group-by-time interaction. Cohen’s d indicates the pooled effect size from baseline to follow-up. Level of significance p<0.05

Outcome	Group	T1 Mean SD	T1 95% CI	T2 Mean SD	T2 95% CI	T3 Mean SD	T3 95% CI	p-value of interaction (group*time)	p-value for the overall trend over time (no grouping)	p-value for difference between groups (regardless the time measured)	Cohen's d (effect size)
PCS	A	21.00 ± 14.09	[10.91, 31.09]	8.80 ± 9.27	[2.16, 15.44]	5.90 ± 7.99	[0.19, 11.61]	0.490	<0.001	0.866	-1.18
	B	21.50 ± 11.23	[13.47, 29.53]	8.60 ± 9.45	[1.84, 15.36]	7.50 ± 9.51	[0.70, 14.31]				
	C	19.60 ± 11.58	[11.32, 27.88]	11.70 ± 12.49	[2.77, 20.63]	11.00 ± 9.80	[3.99, 18.01]				
FABQ	A	31.00 ± 12.64	[21.96, 40.04]	18.70 ± 6.22	[14.25, 23.15]	15.50 ± 6.45	[10.88, 20.12]	0.375	<0.001	0.670	-1.17
	B	36.90 ± 19.95	[22.64, 51.16]	17.70 ± 17.01	[5.54, 29.86]	15.50 ± 14.32	[5.26, 25.74]				
	C	35.50 ± 18.77	[22.07, 48.93]	24.60 ± 12.89	[15.37, 33.83]	20.60 ± 14.32	[10.36, 30.84]				
HADS (Anxiety)	A	6.50 ± 3.72	[3.84, 9.16]	5.70 ± 2.91	[3.62, 7.78]	4.20 ± 3.26	[1.87, 6.53]	0.461	<0.001	0.291	-1.11
	B	7.30 ± 2.71	[5.36, 9.24]	4.70 ± 2.21	[3.12, 6.28]	2.90 ± 2.13	[1.38, 4.42]				
	C	8.50 ± 3.31	[6.13, 10.87]	6.40 ± 2.84	[4.37, 8.43]	5.10 ± 2.56	[3.27, 6.93]				
HADS (Depression)	A	5.80 ± 4.13	[2.84, 8.76]	3.90 ± 3.21	[1.60, 6.20]	4.20 ± 3.36	[1.80, 6.61]	0.874	<0.001	0.533	-0.67
	B	4.60 ± 2.67	[2.69, 6.51]	3.00 ± 2.45	[1.25, 4.75]	2.50 ± 2.59	[0.65, 4.35]				
	C	5.00 ± 3.33	[2.62, 7.38]	3.20 ± 2.53	[1.39, 5.01]	2.50 ± 2.32	[0.84, 4.16]				

Quality of life and global perceived effect

Health-related quality of life, as measured by the SF-12 Physical Component Summary (PCS) and Mental Component Summary (MCS), showed differing patterns over time. PCS scores improved significantly across all groups (p < 0.001 for the main effect of time). The group-by-time interaction was not statistically significant (p = 0.609), and between-group effects, regardless of time, were also non-significant (p = 0.672). For the MCS, no significant change over time was observed (p = 0.242), and both the group-by-time interaction (p = 0.951) and between-group effect (p = 0.075) were not significant. Effect sizes ranged from −0.13 for MCS to 1.20 for PCS.

Post hoc contrasts showed that PCS scores increased significantly from baseline to both post-intervention and follow-up in all groups. No significant between-group differences were found at any time point. MCS scores remained stable across all time points, with no significant changes within or between groups. Summary statistics, confidence intervals, and model results are presented in Table 6.

**Table 6:** SF-12 Physical and Mental Component Summary Scores (PCS and MCS) Across Time Points by the Treatment Group This table presents mean scores for the 12-Item Short Form Health Survey (SF-12) Physical Component Summary (PCS) and Mental Component Summary (MCS) at baseline (T1), post-intervention (T2), and two-month follow-up (T3), stratified by the intervention group. A: Group A, B: Group B and C: Group C. Values are reported as means with standard deviations (SD) and 95% confidence intervals (CI). p-values correspond to fixed effects from linear mixed-effects models evaluating time, group, and group-by-time interaction. Cohen’s d represents the effect size from baseline to follow-up. Level of significance p<0.05

Outcome	Group	T1 Mean SD	T1 95% CI	T2 Mean SD	T2 95% CI	T3 Mean SD	T3 95% CI	p-value of interaction (group*time)	p-value for the overall trend over time (no grouping)	p-value for difference between groups (regardless the time measured)	Cohen's d (effect size)
SF12 PCS	A	35.95 ± 8.42	[29.93, 41.97]	44.46 ± 6.69	[39.67, 49.25]	47.17 ± 4.97	[43.62, 50.73]	0.609	<0.001	0.672	1.20
	B	38.12 ± 9.07	[31.63, 44.61]	45.14 ± 6.42	[40.54, 49.74]	46.11 ± 6.72	[41.30, 50.92]				
	C	40.85 ± 5.33	[37.04, 44.66]	46.38 ± 6.71	[41.58, 51.18]	46.78 ± 6.84	[41.89, 51.67]				
SF12 MCS	A	46.27 ± 5.21	[42.54, 49.99]	45.30 ± 3.08	[43.10, 47.50]	45.25 ± 3.18	[42.98, 47.52]	0.951	<0.001	0.075	-0.13
	B	45.62 ± 4.06	[42.72, 48.52]	45.33 ± 6.03	[41.02, 49.64]	45.27 ± 4.07	[42.36, 48.18]				
	C	48.67 ± 2.82	[46.65, 50.69]	47.03 ± 3.24	[44.71, 49.35]	48.46 ± 3.63	[45.86, 51.06]				

GPE scores showed minimal change over time, with mean scores ranging from 2.90 to 3.40 at follow-up. Neither the main effect of time nor the group-by-time interaction reached statistical significance.

Adherence to exercise

Adherence to prescribed exercise routines was assessed in Groups B and C. Reported adherence improved significantly over time (F= 8.57, p = 0.009). During weeks 1-8, mean adherence was 63.8% in Group B and 70.2% in Group C. In weeks 9-16, adherence increased to 69.6% and 72.3%, respectively. The overall average adherence across the full 16-week period was 66.6% in Group B and 70.8% in Group C. No significant group-by-time interaction was observed (F= 1.87, p = 0.188), and no between-group difference was found (F= 0.24, p = 0.631).

Summary of key findings

The most important outcomes based on statistical significance, clinical relevance, and effect size are presented in Table 7.

**Table 7:** Summary of Key Outcomes This table summarizes time effects, group-by-time interactions, and effect sizes (Cohen’s d) for all outcomes. Time effects indicate overall change; interaction effects reflect differences between groups over time. Effect sizes are interpreted as small (up to 0.2), moderate (up to 0.5), or large (≥0.8). p < 0.05 was considered significant. PPTs were measured in kg/cm² and 5RSTS was measured in seconds. Level of significance p<0.05. PPT: Pressure Pain Threshold; VAS: Visual Analogue Scale; SF-MPQ: Short Form McGill Pain Questionnaire; RMDQ: Roland Morris Disability Questionnaire; 5RSTS: Five-Repetition Sit-to-Stand Test; PCS: Pain Catastrophizing Scale; FABQ: Fear Avoidance Beliefs Questionnaire; HADS: Hospital Anxiety and Depression Scale; SF-12: 12-Item Short Form Health Survey; MCS: Mental Component Summary; GPE: Global Perceived Effect

Outcome	Time Effect (p)	Interaction (p)	Effect Size	Best Response Group
PPT (all sites)	<0.001	0.010–0.094	Cohen’s d = 1.06–2.09	Group B
VAS	<0.001	0.924	Cohen’s d = −3.32	All groups
SF-MPQ Total	<0.001	0.442	Cohen’s d = −1.93	All groups
RMDQ	<0.001	0.296	Cohen’s d = −1.85	All groups
5RSTS	<0.001	0.629	Cohen’s d = 0.57	Group C
PCS	<0.001	0.490	Cohen’s d = −1.17	Group A/B
FABQ Total	<0.001	0.375	Cohen’s d = −1.17	Group A/B
HADS Anxiety	0.005	0.461	Cohen’s d = −1.11	All groups
HADS Depression	0.024	0.874	Cohen’s d = −0.67	All groups
SF-12 PCS	<0.001	0.609	Cohen’s d = 1.2038	Group C
SF-12 MCS	0.242	0.951	Cohen’s d = −0.13	Stable scores across groups
GPE	0.327	0.421	Cohen’s d = 1.27	Same across groups

## Discussion

This pilot trial evaluated the feasibility and preliminary effects of acupuncture alone and in combination with either stretching or modified Qigong in individuals with cLBP. All interventions were well tolerated, with no adverse events or dropouts, and demonstrated high adherence, confirming the protocol’s acceptability and suitability for progression to a fully powered randomized controlled trial.

Clinically meaningful improvements were observed across multiple outcome domains, including pain sensitivity, pain intensity, physical function, psychological distress, and health-related quality of life. Reductions in pain intensity on the VAS (−4.7 points) and SF-MPQ exceeded the established minimal clinically important difference (MCID) of 2 points [31]. Disability scores on the Roland-Morris Disability Questionnaire declined by 7 points, surpassing the MCID of 3.5 points [32]. Physical performance, measured by the 5RSTS, improved by more than 6 seconds, well above the 2.5-second MCID for musculoskeletal populations [18].

Psychosocial outcomes showed similarly meaningful change. Scores on the Pain Catastrophizing Scale and Fear-Avoidance Beliefs Questionnaire decreased by 15.1 and 15.5 points, exceeding reported MCIDs of 8-11 and 13 points, respectively [33,34].

The absence of significant group × time interactions likely reflects the small sample size and limited power of the pilot design. Descriptive trends suggested potential differences between groups, with numerically larger gains in pressure pain threshold and physical function observed in the combination therapy groups. These trends should be interpreted cautiously, given the pilot design and absence of significant interaction effects. However, they are consistent with previous studies proposing additive benefits of multimodal interventions targeting both peripheral and central mechanisms [5,35]. Increases in PPTs in our study consistently exceeded the 17-33% range, which has been suggested in the literature as the MCID for chronic pain populations [36].

Comparative evidence suggests that while acupuncture alone produces clinically significant benefits for cLBP, the incremental advantage of combining it with other modalities is not consistently demonstrated. Djavid et al. found no short-term benefit from adding low-level laser therapy to exercise, with between-group differences emerging only at delayed follow-up [37]. Similarly, another study investigating the feasibility of combining auricular acupuncture with supervised exercise did not observe a definitive additive effect [38]. Mu et al. (2020), in a Cochrane review, concluded that although acupuncture may reduce pain and improve function more than usual care, the evidence on combined or multimodal interventions remains uncertain due to inconsistency and low certainty of evidence [39]. A recent network meta-analysis by Baroncini et al. (2024) supported the use of multimodal strategies but also emphasized heterogeneity and methodological limitations [35]. The descriptive trends in this trial-favoring greater gains in PPT and physical function in the combination groups-align with emerging evidence suggesting a potential additive benefit, though confirmation in larger trials is needed.

The observed improvements were accompanied by large effect sizes, including for pain intensity (Cohen’s d = −3.32), disability (Cohen’s d = −1.84), pressure pain thresholds (Cohen’s d = 0.79-1.05), physical function (d = 0.57), pain catastrophizing (Cohen’s d = −1.18), and fear-avoidance beliefs (Cohen’s d = −1.17). These exceed effect sizes commonly reported in meta-analyses of acupuncture and exercise for cLBP [5,8,40]. While this raises the possibility of additive effects in combination groups, the magnitude of change observed in the acupuncture-only group suggests that substantial benefit may also be attributable to acupuncture alone.

Although no control group (natural course of the disease) was included, this limitation should be viewed in the context of the patient population. cLBP is generally stable over time, and spontaneous recovery is uncommon after 12 weeks’ symptom duration [41]. Therefore, the observed improvements are unlikely to reflect natural history alone.

Adherence to home-based interventions was measured via self-report daily diaries, which may overestimate actual compliance due to recall or desirability bias [42]. Future studies should incorporate objective adherence verification, such as digital tracking or therapist logs. Additionally, no adjustment was made for multiple comparisons. This decision aligns with recommendations for pilot studies designed to identify clinically relevant signals rather than test formal hypotheses [30]. External generalizability was not a primary focus of this pilot study.

## Conclusions

This pilot trial confirmed the feasibility and acceptability of acupuncture-based multimodal interventions for cLBP and demonstrated clinically meaningful improvements in pain, disability, psychological distress, and physical function. These findings support the need for a fully powered trial to evaluate comparative effectiveness and underlying mechanisms in cLBP management.

## Appendices

Appendix A - stretching program (physiotherapy group)

The stretching program was designed to target key muscle groups affecting lumbopelvic mobility and posture in individuals with chronic low back pain. Exercises were supervised by a licensed physiotherapist.

**Table 8:** Stretching Program Exercises Including Prescription Parameters tretching exercises were held for 30 seconds and repeated three times per limb or direction, unless otherwise indicated. Repetitions were used for mobility and activation exercises. Rest between repetitions was 30 seconds. Each session lasted approximately 30 minutes and was performed twice per week for five weeks. Exercises were adapted individually based on participant comfort and clinical presentation. QL = Quadratus Lumborum.

Exercise	Target Muscle(s)	Prescription
Supine single knee to chest	Lumbar paraspinals	3 × 30 seconds per leg
Supine double knee to chest	Lumbar and gluteals	3 × 30 seconds
Supine piriformis stretch	Piriformis	3 × 30 seconds per leg
Supine hamstring stretch with towel	Hamstrings	3 × 30 seconds per leg
Standing gastrocnemius stretch	Calf	3 × 30 seconds per leg
Standing iliopsoas stretch	Hip flexors	3 × 30 seconds per leg
Standing trunk flexion with hands on thighs	Thoracolumbar extensors	3 × 30 seconds
Seated lateral trunk flexion	Obliques/QL	3 × 30 seconds per side
Supine pelvic tilts	Core activation	3 sets of 10 repetitions
Cat–camel mobilization	Spinal mobility	3 sets of 10 repetitions

Appendix B - modified Qigong program (Qigong group)

The Qigong intervention followed a clinically adapted version of the Baduanjin (Eight Sections of Brocade) form, delivered under physiotherapeutic supervision. The movements practiced were: (1) Raise both hands to regulate the internal organs, (2) Draw the bow to shoot the eagle, (3) Separate heaven and earth, (4) Wise owl gazes backward, (5) Sway the head and shake the tail, (6) Touch the toes to strengthen the kidneys and waist, (7) Clench the fists and glare fiercely, and (8) Bouncing on the toes.

The program consisted of 15-20-minute sessions, conducted twice weekly under supervision during the first four weeks. From week five onward, participants were encouraged to continue with home practice using written instructions. All sessions were supervised by a physiotherapist trained in therapeutic Qigong. The exercises were selected and adapted in consultation with Dr. Wen Zhong (Shanghai University of Traditional Chinese Medicine) to ensure appropriateness for individuals with chronic low back pai

Note: Although described as a “modified” version, specific biomechanical adjustments (e.g., range restrictions) were made according to participant tolerance, with general avoidance of excessive lumbar flexion/extension or rotation.

Appendix C - standardized procedure and training for pressure algometry

This appendix outlines the protocol for training the assessor and administering pressure pain threshold (PPT) measurements using a digital algometer in accordance with established methodological standards for clinical pain research.

C1. Training and Standardization of the Assessor

Prior to participant assessments, the researcher responsible for pressure algometry underwent formal training to ensure methodological reliability and consistency. Competency was verified by two independent evaluators with prior research experience using pressure algometry. To be certified for data collection, the assessor was required to apply pressure at a consistent rate of 1 kg/sec, maintain 10-second rest intervals between repeated measures at the same site, and reliably identify all measurement sites on at least five healthy volunteers. Training was conducted using the JTech Echo Commander digital algometer-the same device used in the main study-and emphasized inter-rater agreement, consistency of technique, and adherence to the predefined measurement protocol.

C2. Measurement Procedure

All PPT assessments were performed using the JTech Echo Commander digital algometer at three standardized time points: baseline (T1), post-intervention (T2), and two-month follow-up (T3). The same assessor conducted all measurements to minimize inter-rater variability. 

The measurement protocol involved applying pressure at a constant rate of 1 kg/sec with a 10-second interval between successive measurements at each site. Pressure pain threshold was assessed at nine anatomical locations: the spinous process of L3; the left and right paraspinal muscles at L4 and L5; the left and right gluteus medius; the left and right tibialis anterior; and the left and right trapezius. To control environmental variables, all sessions were scheduled at similar times of day for each participant. Participants were instructed to abstain from caffeine, alcohol, nicotine, intense physical activity, and analgesic medication on assessment days, as these factors could affect pain sensitivity. The testing sequence was standardized across all time points to ensure procedural consistency.
